# Evaluating the Influence of Acute and Chronic Orofacial Pains on the Overall Comprehensive Quality of Life

**DOI:** 10.7759/cureus.63625

**Published:** 2024-07-01

**Authors:** Akula Madhavi, Mekala Mary Sujatha, Muthahera Mazhar, Kankshini Pabba, G Lavanya, Amrita Gupta

**Affiliations:** 1 Department of Conservative Dentistry and Endodontics, Sai Dental Surgery, Hyderabad, IND; 2 Department of Oral Medicine and Radiology, Government Dental College and Hospital, Vijayawada, Vijayawada, IND; 3 Department of Family Dentistry, Willow Knolls Family Dental, Peoria, USA; 4 Department of Biomedical Science, Rutgers University - Newark, Newark, USA; 5 Department of Oral Medicine and Radiology, Malla Reddy Dental College for Women, Hyderabad, IND; 6 Department of Oral Medicine and Radiology, Terna Dental College and Hospital, Navi Mumbai, IND

**Keywords:** persistent dentoalveolar pain disorder, trigeminal neuralgia, atypical dental pain, temporomandibular disorders, injury, quality of life, chronic orofacial discomfort, acute orofacial discomfort

## Abstract

Background

Orofacial discomfort refers to various disorders that affect the mouth, jaws, and face. These conditions may substantially influence a person's quality of life (QoL). This kind of pain may be categorised into two primary classifications: acute and chronic. Acute orofacial pain (OFP) usually occurs suddenly and lasts for a short period. It is commonly caused by specific factors such as dental treatments, traumas, or infections. Hence, this study aimed to assess the influence of acute and chronic orofacial discomfort on the overall QoL.

Methodology

This research used a convenience sample to gather data from the five groups. It was conducted as a cross-sectional study. Four categories of OFP syndromes were utilised: temporomandibular disorders (TMDs), atypical dental pain (ADP), trigeminal neuralgia (TN), and persistent dentoalveolar pain disorder (PDAP). In addition, a control group consisting of individuals who did not experience any discomfort was also included in the study. Participants received a standardised explanation of the questionnaires utilised, and in most instances, they completed them at the clinic under the supervision of one of the investigators.

Results

The correlations between each version of the Oral Health Impact Profile (OHIP) were statistically significant (p < 0.001), with correlation values ranging from 0.92 to 0.97. When comparing the occurrence of OHIP items across the four pain types, we identified 18 variations that exceeded the 35% prevalence threshold we established for identifying important items that frequently occur out of the 315 comparisons. The question "Have you had a painful aching in your mouth?" showed a low frequency in patients with TN, TMD, and ADP but a significant prevalence in patients with PDAP (90%). This difference in prevalence across pain types was the biggest seen. The question that seemed to distinguish between the pain categories most effectively was "Have you experienced a toothache?" This question had a high occurrence in PDAP (65%) and ADP (60%), perhaps allowing for differentiating these two diseases from TN and TMD. The highest prevalence differences, over 30%, were most often seen when PDAP was included as one of the disorders being compared. ADP had the fewest variances, over 30%, occurring just seven times.

Conclusion

Both acute and chronic OFPs have a major negative influence on QoL, but they affect it differently and to differing extents. Injury-related acute pain obviously poses rapid and severe restrictions on physical function and causes temporary psychological distress and temporary social exclusion. On the other hand, chronic pain for the patient always implies being locked in a physical world that does not allow him or her to bypass physical limitations, psychological disorders remain constant, and isolation from other people persists for life.

## Introduction

Focusing on their effect, orofacial pain (OFP), meaning any type of pain occurring in the face and mouth regions, significantly impacts individuals’ prevailing activity. This pain may be acute or chronic, and depending on the duration and specific complications, it can cause certain problems and affect the quality of life (QoL) of an individual. The temporary nature of the facial pain, with instances being very sharp and often linked to certain provoking factors such as operations or injuries in the oral cavity, can significantly impact daily life. Further, OFP is classified as acute when the pain subsides after days to weeks or less than six months and may emanate from conditions like acute temporomandibular disorders (TMDs), acute trigeminal neuralgia (TN), or acute idiopathic facial pain [1]. The International Association for the Study of Pain (IASP) defines pain as an unpleasant sensory and emotional experience associated with actual or potential tissue damage. The ache lasts longer than expected for recovery or when there is a failure to attain the expected QoL [2]. There is evidence that various initial psychological variables can predict the outcome of chronic pain following surgery. QoL may be defined broadly, encompassing several domains like physical health, psychological state and functioning, and social relationships. Sudden, sharp, severe pain in the facial front area can incapacitate the patient, making it impossible to eat, speak, or perform other routine functions. Pain is chronic when it lasts for a long time and may take the form of constant suffering, which may be complicated by changes in eating habits, sleep interruption, and fatigue. Long-term OFP has been found to result in greater extents of physical disabilities and deterioration in health compared to short-term OFP. Pain concern is episodic and may cause tension; chronic pain is characterised by moderate to severe psychological outcomes, including depression, anxiety, and reduced QoL. Because the condition is long-term, it may foster feelings of helplessness and hopelessness in the chronic pain sufferer.

Chronic pain, as a result, manifests significant psychological distress and lower total mental health compared to others [1,3]. Both short-term and long-term OFPs may limit interpersonal interactions with others and, in turn, alter workplace productivity. Short-term pain flare-ups may lead to temporary work or social activity exemptions, while chronic pain leads to unemployment and seclusion. Long-term pain frequently creates a low desire for social participation and fewer social activities, alongside significant changes in interpersonal relationships. Thus, poor social functioning is manifest [4]. Individuals suffering from long-lasting pain often experience a shift in their beliefs and thought processes, which, in turn, impact their emotional and cognitive pathways. These pathways play a role in how they perceive and experience pain. Over time, persons suffering from chronic pain may see a decline in their capacity to operate at their best, and some may choose to retire from work prematurely [5]. Chronic pain disorders that do not involve the mouth or face might result in a considerable level of impairment. In the United States, they account for 21% of visits to accident and emergency rooms and contribute to 25% of yearly job absenteeism, resulting in a large increase in the economic burden. OFP is directly associated with higher levels of daily productivity loss and overutilisation of healthcare resources [6,7]. The occurrence rate of OFP varies from 17 to 26%, with up to 11% classified as chronic orofacial pain (COFP) [8]. COFP is often connected to psychological illnesses, and there is a significant correlation between persistent OFP and feelings of sadness and anxiety, leading to reduced psychological functioning. Without recognising psychological components, pain treatment is restricted, and the rehabilitation process is often hindered due to variations in an individual's psychological predisposition, leading to various reactions to pain [9-12].

## Materials and methods

Study design, setting and participants

This research used a convenience sample to gather data from five distinct groups and was conducted as a cross-sectional study. Four categories of OFP syndromes were utilised: TMDs, atypical dental pain (ADP), TN, and persistent dentoalveolar pain disorder (PDAP). Additionally, the "non-comfort-seeking" group, who reported no dissatisfaction, was incorporated into the study. Participants were provided with a uniform explanation regarding the questionnaires used in the study, and in most cases, the questionnaires were completed at the clinic under the guidance of one of the investigators. Consent was obtained or waived by all participants in this study. Sai Dental Surgery, Hyderabad, India, issued IEC/SDS/2023/EDNDO approval.

The study sample, therefore, comprised all patients aged 18 years and above who were capable of responding to questions and met the selection criteria. Patient samples with TMD were diagnosed according to the diagnostic criteria (DC) for TMDs. Diagnoses of myalgia, myofascial pain, arthralgia, and headache associated with TMD were made in the process. Persistent/irreversible pulpitis and/or symptomatic apical periodontitis were diagnosed according to Gutmann et al.'s DC set for the 3R condition. TN was diagnosed using the International Classification of Headache Disorders, 2nd edition (ICHD-II) DC for Classical Trigeminal Neuralgia. Individuals suffering from chronic dentoalveolar pain were diagnosed by a skilled physician, qualified by a professional board, using the DC outlined by Nixdorf et al. [13]. The pain-free controls were required to answer "NO" to the following question: "Have you experienced any discomfort in your face, mouth, teeth, jaw, or ears within the past three months?" Participants who had not sought dental treatment over the last three months were included in this research.

Selection criteria

The study excluded participants who had previously experienced traumatic injuries to the orofacial region, participants with significant systemic illnesses that affected pain sensitivity, such as fibromyalgia or widespread bodily pains, and participants who had undergone TMJ surgery or intra-articular steroid injection.

Data sources and variables

The OHIP-49, devised by Slade and Spencer in 1994 [14], consists of 49 items. Although there are shorter variants of the assessment with 14 or 5 questions and versions specialised for certain conditions like TMDs, we decided to use the original form, OHIP-49, since it provides the most thorough evaluation of overall oral health-related QoL (OHRQoL). Subjects are asked to assess the frequency of their encounter with each of the 49 items on a five-point unipolar ordinal scale. The scale ranges from zero, indicating "never," to four, indicating "very often." In this research, we used both a one-month reference period and included the term "jaws" in the OHIP-49 version that was used.

Due to methodological problems in the domain scores, we chose not to analyse the seven domains of the OHIP-49. Instead, we utilised the basic summary score suggested by the Dimensions of Oral Health-Related Quality of Life (DOQ) study. In this research, we use the straightforward sum of the answer codes of the 49 items as our measure of OHRQoL.

Statistical analysis

The data were analysed using IBM SPSS Statistics for Windows, Version 25 (Released 2017; IBM Corp., Armonk, NY, United States). The mean OHIP-49 summary scores of the four conditions and their 95% confidence intervals were compared to those of the control group to evaluate the particular effect of each condition on OHRQoL. Additionally, they were compared to evaluate whether there are variations in OHRQoL impairment across various OFP situations. After evaluating the extent of score disparities, we conducted a statistical analysis using analysis of variance (ANOVA) to examine differences in mean scores. If the overall ANOVA test yielded significant results, we performed t-tests for pairwise comparisons. The impact on OHRQoL was assessed by extracting the 14 items from the OHIP-14 questionnaire and the five items from the OHIP-5 questionnaire derived from the OHIP-49. The average summary score for each questionnaire was calculated to facilitate comparison with other studies using different OHIP metrics.

## Results

Subject attribute Table 1 presents the characteristics of the sample in relation to age and gender. Considering the specific characteristics of the pain syndromes that were intentionally researched, there were noticeable variations in age and gender, which align with the typical progression of the diseases under investigation. The average OHIP summary scores for each group analysed are also included in Table 1. The correlations between each version of the OHIP were statistically significant (p < 0.001), with correlation values ranging from 0.92 to 0.97.

**Table 1:** Basic parameters of the participants OHIP: Oral health impact profile; TMDs: Temporomandibular disorders; ADP: Aypical dental pain; TN: Trigeminal neuralgia; PADP: Persistent dentoalveolar pain disorder; N: Number of participants

Parameter	TMD (N = 50)	ADP (N = 50)	TN (N = 20)	PDAP (N = 20)	Controls (N = 20)
Gender					
Male - N (%)	10 (20%)	20 (40%)	6 (30%)	2 (10%)	8 (40%)
Female - N (%)	40 (80%)	30 (60%)	14 (70%)	18 (90%)	12 (60%)
Age in years (N)	45 (17)	48 (12)	60 (18)	52 (12)	45 (14)
OHIP-14 (mean ± standard deviation)	20.03 ± 2.46	17.08 ± 1.39	18.96 ± 2.67	21.12 ± 2.48	1.55 ± 0.56
OHIP-49 (mean ± standard deviation)	63.12 ± 4.65	56.87 ± 6.65	57.79 ± 7.46	70.76 ± 6.47	7.88 ± 1.98
OHIP-5 (mean ± standard deviation)	7.77 ± 0.69	7.11 ± 0.37	6.99 ± 0.42	8.07 ± 0.98	0.95 ± 0.04

The whole range of ratings for the four pain conditions is in contrast to the control group. The effect of pain conditions was significantly different compared to controls (p < 0.001 in all comparisons), although there were no significant differences across the various pain situations. The median and various quantiles of score distributions were comparable across the different situations. There was no discernible trend indicating that the score distribution of a certain condition consistently had higher or lower quantiles than other distributions. Table 2 illustrates the distinctions between the control, pain state, and inter-pain condition. The disparity between the control and pain conditions groups was a minimum of 35 points on the OHIP-49 scale. A negative value indicates that the OHIP score for the row condition is less than the OHIP score for the column condition. 

**Table 2:** OHIP score differences and comparisons of four orofacial pain conditions and control OHIP: Oral health impact profile; TMD: Temporomandibular disorders; ADP: Aypical dental pain; TN: Trigeminal neuralgia; PADP: Persistent dentoalveolar pain disorder The values denote distinctions between the control pain state and the inter-pain condition with the corresponding ranges in the brackets

		PDAP	TN	TMD	ADP
Controls	OHIP-49	63.34 (78.98 to 45.47)	51.43 (68.47 to 32.36)	55.47 (72.12 to 31.43)	49.76 (60.87 to 33.23)
	Cohen’s d	2.77 (3.76 to 2.32)	2.12 (2.54 to 1.65)	1.89 (2.78 to 1.47)	2.43 (3.42 to 2.15)
PDAP	OHIP-49	–	12.46 (11.68 to 31.45)	8.37 (12.67 to 22.26)	15.12 (1.98 to 28.87)
	Cohen’s d	–	0.53 (0.18 to 1.45)	0.31 (0.24 to 0.88)	0.5 (0.1 to 1.0)
TN	OHIP-49	–	–	4.56 (23.43 to 14.23)	2.5 (14.7 to 19.7)
	Cohen’s d	–	–	0.17 (0.76 to 0.54)	0.13 (0.33 to 0.99)
TMD	OHIP-49	–	–	–	5.34 (7.12 to 18.87)
	Cohen’s d	–	–	–	0.33 (0.14 to 0.78)

Therefore, it surpasses the minimum important difference (MID) of six OHIP-49 points. The disparities in pain conditions varied significantly, with the greatest mean score difference between PDAP and ADP, and the lowest difference observed between TN and ADP. Except for the disparity between TN and ADP and TN and TMD, all other values surpassed the MID. Using Cohen's d-effect sizes, standardised differences were used to compare groups, revealing a range of impact magnitudes (Table 2). The effect sizes for pain conditions were moderate (n = 1), modest (n = 3), and non-existent (n = 2). This discovery reinforces the trend of slight variations arising across pain types, even when a large range of uncertainty surrounds the specific estimations.

The degree of impact between the chronic pain conditions (TMD, TN, PDAP) and the acute pain (ADP) group did not show a statistically significant difference, as determined by the two-sample t-test (p = 0.33). An impact size of 0.19 was observed between the chronic and acute pain groups. When comparing the occurrence of OHIP items in the four pain types, we found 18 variations in the 315 comparisons higher than the 35% prevalence threshold we established for important items that occur often (Table 3). Except for two occurrences of the item "Worried by dental problem" and the item "Tense because of problems with your teeth, mouth, denture or jaw," most regularly appearing items focused on different aspects of dental, oral, and OFP.

**Table 3:** OHIP items OHIP items demonstrating differences in prevalence greater than 30% for frequent impacts ("fairly often" or "very often") among possible 315 pairwise comparisons for six pairings of four orofacial pain conditions and 49 OHIP items OHIP: Oral health impact profile; TMDs: Temporomandibular disorders; ADP: Aypical dental pain; TN: Trigeminal neuralgia; PADP: Persistent dentoalveolar pain disorder

Item	Pair of conditions	Item prevalence condition 1 (%)	Item prevalence condition 2 (%)	Difference in prevalence (%)
Have you had a distressing sensation of discomfort in your oral cavity?	PDAP-TN	90	35	55
	PDAP-ADP	90	50	45
	PDAP-TMD	90	55	35
Have you experienced any discomfort or pain in your jaw?	TMD-TN	70	30	45
	TMD-PDAP	65	35	35
	ADP-TMD	70	25	45
Have you had a headache as a result of issues with your teeth, oral cavity, jaw, or dentures?	TMD-TN	60	5	50
	TMD-ADP	60	10	45
	PDAP-TN	40	5	40
Have you had dental pain?	ADP-TMD	60	25	35
	PDAP-TN	65	20	50
	PDAP-TMD	65	25	40
Have you had any concerns or anxieties related to issues with your jaw or dental health?	TMD-ADP	60	30	30
	TMD-TN	55	25	30
Are you experiencing distress due to dental issues?	PDAP-TN	70	35	40
	PDAP-ADP	70	35	35
Experienced a state of tension?	PDAP-TN	55	20	30
Struggling to unwind?	PDAP-ADP	55	20	30

The question "Have you had a painful aching in your mouth?" showed a low frequency in patients with TN, TMD, and ADP but a significant prevalence in patients with PDAP (90%). This difference in prevalence across pain types was the biggest seen. The question that seemed to distinguish between the pain categories most effectively was, "Have you experienced a toothache?" This question had a high occurrence in PDAP (65%) and ADP (60%), perhaps allowing for differentiating these two diseases from TN and TMD. The highest prevalence differences, over 30%, were most often seen when PDAP was included as one of the disorders being compared. ADP had the fewest variances, over 30%, occurring just seven times. The same has been depicted in Table 3.

## Discussion

Whether acute or chronic, orofacial discomfort substantially influences patients' QoL, impacting several aspects such as physical health, mental well-being, and social functioning. Comprehending these effects is essential for healthcare practitioners to formulate complete treatment strategies, including pain alleviation and the wider elements of patient care. Physical impact, such as acute OFP, which is often caused by dental treatments, trauma, or infections, generally leads to quick and significant suffering. This can result in challenges when carrying out fundamental tasks such as consuming food, communicating verbally, and upholding oral cleanliness. Acute pain may greatly interrupt daily routines and decrease overall functioning due to immediate physical restrictions.

The features hate sharp, sudden, and severe pains that stir considerable worry, anxiety, and dread. These psychological reactions may escalate the degree of pain, thus developing a negative cycle of suffering which prevents an individual from managing pain to a considerable extent. It has been established that there is a high correlation between pain and depression, anxiety, and chronic stress. Suffering from chronic pain, which is long-lasting and inevitably recurrent, a patient feels helpless and frustrated, which significantly decreases the quality of psychological health. The latter would define how New Zealand patients experience a diminished health status and life satisfaction, struggling with emotional distress from chronic pain.

These findings show that four types of OFP - TMD, APD, TN, and PDAP - have impacted OHRQoL. In analysing the data concerning the influence of four types of OFP on the OHIP-49, a slight variation was established concerning the OHIP-5 subset wherein the degree of impairment was analysed. In particular, the estimated impairment for the participants with TN was the lowest based on the results of the OHIP-49 and OHIP-14 subscales.

This might be because the above instrument’s sensitivity decreases when the number of elements is reduced. This statistic is beneficial in a way that it shows how values differ across question numbers. Also, it has available data that other researchers can utilise when comparison is desirable and when planning future sample sizes in a study. This study's results align with previous studies that used the OHIP-49 questionnaire for patients with TMDs. Despite this, it is quite challenging to compare our findings directly with prior studies that compared ADP with OHIP since the researchers employed a wide range of methodologies [15-18]. Since the full OHIP-14 questionnaire was not used, the OH scale research team derived the 14 items from the OHIP-49 questionnaire. That said, it is possible to align the study results with other works that included the OHIP-14 in patients with TMD and ADP [17-19]. Given that the short variation of the OHIP positively correlated with pain with both the OHIP-49 and OHIP-14, it is evident that the short form of the OHIP does indeed measure the overall influence of pain on OHRQoL. OHIP-14 and OHIP-7 are important to note that the shorter versions were not provided directly to the participants; rather, they are abbreviated forms of the OHIP-49. Despite these results, these must be reproduced using the actual measures to know, for instance, whether the said outcomes would change. Pertaining to practical experience or observation, this research has focused precisely on the variation of items to the assessment of different pain groups to establish an empirical yardstick for measuring how this pain condition impacts people's lives. In relation to this issue, fluctuations in incidence were more often observed between laryngocele and PDAP compared to other disorders.

This indicates that PDAP has exerted a slightly higher impact on the changes throughout the analysed period, while the effect of TMD, TN, and ADP totals. Finally, in general, the present trend was not very robust, and therefore it may be argued that the findings of the present research could be fraught with errors because of a relatively small number of participants and large confidence intervals.

It was expected that people with TN would have a higher OHIP-49 score because of the nature of the disease as a severe pain sickness; however, results show that patients with TN had a similar OHIP-49 score as patients with other kinds of OFP problems. A potential rationale for this result may lie in the inability of the OHIP-49 questionnaire to capture the episodic and relapsing nature of TN adequately. If there is no sign or occurrence of that particular disease for a given period prerequisite by the OHIP, then the affected person may be unable to show any impacts or implications of the disease. This suggests that there may be a possibility for patients to be inclined to recall and report the earlier painful intensity inelastically when they want to fill in the OHIP-49. Hence, further exploration of the intended relationship of this memory bias is crucial in future research.

One common cause that may have a bearing on the results discovered in TN is that, although most of the patients complained of pain, they might have already begun undergoing therapy before their consultation. It might have lessened the intensity and/or frequency of the pain they were experiencing then. While there may have been some factors that could have contributed to the findings, it is evident from this study that this condition affected the QoL of the sufferers. Thus, these results are in line with past research that has employed other indicators to address the same concern [20].

The literature has not before reported on the assessment of the biopsychosocial effect of TN and PDAP using an OHRQoL score. The average OHIP-49 summary score for PDAP individuals was elevated, indicating a diminished QoL. Other studies have examined the effect of PDAP using different approaches and shown that patients with PDAP have a substantial psychological and social burden, comparable to what we have shown in our present study [21]. In the only longitudinal trial that tracked PDAP individuals for a maximum of seven years, findings indicated that only one-third of the PDAP people had a reduction in their symptoms [22]. Therefore, based on our results, a considerable number of PDAP patients may suffer from a long-lasting effect on their QoL. The present research findings indicate that the damage caused by PDAP and ADP is comparable. Consequently, patients with PDAP have a persistent and unresponsive "toothache" that is resistant to most forms of therapy. Additional research is necessary to enhance the management of PDAP and mitigate its significant effect. Combining our primary results with our exploratory analyses, we have formulated several theories due to the small variations in OHRQoL across OFP disorders. Firstly, OFP issues often occur together, meaning that pain conditions commonly present simultaneously. For instance, individuals diagnosed with TN or PDAP often have TMD as well. OFP may sometimes radiate to adjacent regions, manifesting as mandibular discomfort. On the other hand, TMD might sometimes be called the dental region. The simultaneous presence of OFP problems, even if one disease is more prominent, leads to a comparable effect of pain across all conditions, as we have discovered.

Furthermore, the documentation of dental, oral, and OFP problems is grouped. They constitute a specific component of the OHRQoL, a significant and separate aspect within the broader concept of OHRQoL. These quantitative results are supported by qualitative findings that indicate the need for a separate dimension for pain in the stomatognathic system rather than including it in the present "physical pain" category of the OHIP-49 [23,24]. This phenomenon may be attributed to several aches being closely related and occurring simultaneously, significantly influencing the OHRQoL.

Limitations

This study has several limitations that should be acknowledged. Firstly, using a convenience sample may introduce selection bias, as participants who are more readily available or willing to participate might not represent the broader population suffering from OFP. Secondly, the study's cross-sectional design limits the ability to infer causality or observe changes over time. Longitudinal studies would be more effective in understanding orofacial discomfort's progression and long-term impacts on QoL. Additionally, the reliance on self-reported questionnaires can lead to response bias, where participants may underreport or overreport their symptoms and their impact due to recall or social desirability bias. The study also did not account for potential confounding variables such as socioeconomic status, access to healthcare, or psychological factors, which could influence both the perception of pain and QoL. Due to methodological problems in the domain scores, we chose not to analyse the seven domains of the OHIP-49. Instead, we utilised the basic summary score suggested by the DOQ study. Finally, categorising OFP into only four types may oversimplify the complex nature of these conditions, and future research should consider a more nuanced classification that includes other relevant subtypes and contributing factors.

## Conclusions

Both acute and chronic OFPs have a major negative influence on QoL, but they affect it differently and to differing extents. Injury-related acute pain poses rapid and severe restrictions on physical function and causes temporary psychological distress and social exclusion. On the other hand, chronic pain for the patient always implies being locked in a physical world that does not allow him or her to bypass physical limitations, psychological disorders remain constant, and isolation from other people persists for life. While aiming at managing OFP, it is helpful to establish an approach that goes beyond the physical aspect of pain management and considers the patient’s psychological and social state.

Concerning acute and chronic health difficulties, clinicians should be highly attentive to the timely and accurate recognition of diseases, successful pain interventions, and comprehensive, dynamic psychosocial interventions. In this manner, they can partly contribute to minimising the extensive impacts of OFP and significantly improve patients' lifestyles.
